# Housing Poverty and Healthy Aging in China: Evidence from the China Health and Retirement Longitudinal Study

**DOI:** 10.3390/ijerph18189911

**Published:** 2021-09-21

**Authors:** Peng Nie, Yan Li, Lanlin Ding, Alfonso Sousa-Poza

**Affiliations:** 1 School of Economics and Finance, Xi’an Jiaotong University, Xi’an 710061, China; liyan4119119024@stu.xjtu.edu.cn (Y.L.); dinglanlin@stu.xjtu.edu.cn (L.D.); alfonso.sousa-poza@uni-hohenheim.de (A.S.-P.); 2 Institute for Health Care & Public Management, University of Hohenheim, 70599 Stuttgart, Germany

**Keywords:** housing quality, housing quantity, housing poverty, healthy aging, China

## Abstract

Background: Although prior research on the housing–health linkage suggested that those with poor housing conditions are more likely to report poor health, it is dominated by Western studies and offers little evidence on the housing–health relation in China. Scarce is empirical evidence on the potentially detrimental impact of either qualitative or quantitative housing poverty on health outcomes, especially for seniors in China. This paper aims to fill this void by using data from the 2011–2015 China Health and Retirement Longitudinal Study (CHARLS) to provide a comprehensive analysis of the demographic, socioeconomic, and behavioral factors that contribute to changes in healthy aging among Chinese adults aged 60 and over. Methods: Data collected from 8839 adults aged 60 and over in the 2011 and 2015 CHARLS (3732 in 2011 and 5107 in 2015) were used. We first used six blood-based biomarkers to construct a composite measure of the Chinese Healthy Aging Index (CHAI, ranging from 0 (healthiest) to 12 (unhealthiest)) and then assessed the psychometric properties of the CHAI score, including acceptability, internal consistency, convergent validity, discriminative validity and precision. In addition, we employed both mean-based Blinder–Oaxaca and unconditional quantile regression decomposition to decompose the change in healthy aging within the 2011–2015 period. Results: We overall identified a decrease in CHAI score from 5.69 in 2011 to 5.20 in 2015, which implies an improvement in healthy aging during this period. Our linear decomposition revealed that dependent on the type of measure used (whether quality, quantity, or combined quality–quantity), housing poverty explained 4–8% of the differences in CHAI score. Our distributional decompositions also highlighted an important role for housing poverty in the change in healthy aging, accounting for approximately 7–23% of the explained portion. Within this latter, the relative contribution of housing quantity and quality poverty was more pronounced at the median and upper end of the CHAI distribution. We also found household expenditure to be significantly associated with healthy aging among older Chinese adults and made the largest contribution to the improvement in healthy aging over time. Conclusions: The association between housing poverty and CHAI is independent of household expenditure. Regardless of type, housing poverty is positively associated with a decrease in healthy aging. Thus, improved housing conditions boost healthy aging, and housing amelioration initiatives may offer the most effective solution for augmenting healthy aging in China. Improvement of flush toilets and the access to potable water and a separate kitchen require particular attention. Since high-density congested housing has a negative impact on healthy aging, more attention can also be paid to improvements in the available space for older people. Especially at an institutional level, the government may extend the housing policy from a homeownership scheme to a housing upgrading scheme by improving housing conditions.

## 1. Introduction

The global concern of population aging poses a special challenge for the United Nations’ Sustainable Development Goals (SDGs), especially SDG 3, which aims to ensure healthy lives and wellbeing at all ages [1]. Such aging is particularly rapid in China, which has the world’s largest population of adults aged 60 and over [2,3,4], with the 2019 number of approximately 254 million (18.1% of the total population) [5] expected to double by 2050 [6]. Such unprecedented numbers, especially in developing countries such as China, have magnified the importance of healthy aging, “of developing and maintaining the functional ability that enables well-being in older age” [7] (p. 28). One major social determinant of this wellbeing is housing [8], a primary contributor to achieving SDGs, especially SDG 3 [9]. Nevertheless, despite a large body of literature on the housing–health linkage in developed regions such as the United States [10,11,12], Europe [13,14,15,16,17,18,19], New Zealand [20,21], and Australia [22,23,24], evidence in developing countries remains scarce. Housing remains one of the most understudied aspects of aging in China [25].

The case of China is made particularly interesting by its socioeconomic and cultural profiles, beginning with its unprecedented economic growth since the 1978 Reform and Opening-Up Policy, which engendered a remarkable increase in per capita GDP from 385 CNY in 1978 to 70,725 CNY in 2019 [26]. Nonetheless, although the urbanization rate increased from 18% to 61% over the same four decades, these developments have not been accompanied by equal improvements in health [27]. That is, despite an increase in Chinese life expectancy from 66 years in 1978 [28] to 77 years in 2019 [26], health improvements have stagnated [27,29], leading to an increase in widespread health disparities, particularly among older adults [30]. These latter have also emerged as a population for whom poverty is a notable social problem [31]. At the same time, the housing market in China has undergone a fundamental transformation [32,33], shifting from the domination of public-housing rentals to an extremely high rate of homeownership, up from 28% in 1993 to 91% in 2013 [34], one of the highest percentages in the world [35].

The importance of housing conditions in the daily life of older Chinese adults is augmented by a cultural (and anti-institutionalization) preference for aging at home, which has given rise to the stigmatization of nursing homes [36]. Yet, despite significant improvements in average housing conditions, not every household has benefited equally from housing reforms, giving rise to housing inequality [37], especially among seniors [38]. This inequality has also been exasperated by the more than doubling of housing prices between 2007 and 2014 [39]. Because aging in place is a strongly advocated policy initiative for dealing with a rapidly aging population [40], understanding the potential role of housing poverty in healthy aging is important to developing age-friendly living environments for older Chinese adults.

Although a broad body of existing research on the housing–health linkage in Western countries provided overwhelming evidence that poor housing conditions (e.g., no access to hot water, overcrowding, darkness, lack of adequate heating facilities) have a detrimental effect on health, it focuses almost exclusively on developed nations (see Section 1). This focus also dominated a second strand of literature on the homeownership-health nexus [22,23,24,41,42,43,44], which documented the positive influence of homeownership on different health measures, as well as the negative impact of housing instability (e.g., homelessness) and high accommodation costs [23,41].

Evidence for the housing–health relation in China, however, is extremely sparse, with only three easily identifiable relevant studies. In the first, after applying the six-item Kessler Psychological Distress Scale to data from the 2009 Twelve City Migrant Survey, Li and Liu [45] found no association among Chinese migrants between poor housing conditions and perceived stress. Conversely, Wang et al. [46] showed a clear association between better housing conditions and improvement in both physical (based on self-assessed BMI and daily activity limitations) and self-reported health (based on 2010 China Family Panel Study data). More recent work by Chung et al. [47] using a sample of Hong Kong residents also documented a negative impact of lower housing affordability on both physical and mental health.

Hence, although prior research on the housing–health linkage suggests that those with poor housing conditions and house renters (vs. house owners) are more likely to report poor health, it is dominated by Western studies and offers little evidence on the housing–health relation in China. Scarce is empirical evidence on the potentially detrimental impact of either qualitative or quantitative housing poverty on health outcomes, especially for seniors in China. This paper contributes to the literature on housing–health relations in three ways: First, given China’s unprecedented economic growth, accelerating population aging, and unique housing market, our study is the first to investigate the housing poverty-healthy aging link in China using nationally representative data. Based on the existing literature, we aim to test the hypothesis that both qualitative and quantitative housing poverty are positively associated with a decline in healthy aging. Second, by focusing simultaneously on the effects on healthy aging of both qualitative and quantitative housing poverty, we paint a far broader picture of the housing–health linkage. In particular, by combining mean-based Blinder–Oaxaca (BO) and unconditional quantile regression (UQR) decomposition, we are able to quantify housing poverty’s relative contribution to healthy aging changes over time while also assessing its heterogeneous contributions at different quantiles on the unconditional marginal distribution of our Chinese Healthy Aging Index (CHAI) scores.

## 2. Materials and Methods

### 2.1. Data

The data were drawn from the CHARLS, a nationally representative survey of the middle-aged and elderly in China [48], which was co-administered by the National School of Development and the Institute for Social Science Surveys at Peking University. One of a group of aging surveys worldwide, CHARLS was harmonized with the US Health and Retirement Study (HRS), the English Longitudinal Study of Aging (ELSA), and Europe’s Survey of Health, Aging and Retirement (SHARE). The survey, which employed a multistage (county/district, village/community, household) stratified probability proportional to size (PPS) sampling design [48], comprised a national baseline survey conducted in 2011–2012 on 17,708 residents of 10,257 households in 450 villages and urban communities, with three follow-up interviews in 2013, 2015, and 2018 [49]. The CHARLS encompassed one person per household aged 45 or older and their spouse. The sample procedure included three main stages: In the first stage of the survey, all county-level units were stratified by region, within the region by urban district or rural county and by GDP per capita [48]. A total of 150 counties or urban districts were selected with PPS. The second stage involved the random selection of 3 primary sampling units (villages and urban neighborhoods) with PPS at each county-level unit [48]. In the final stage, a short screening form was employed to identify whether the household had a member who met the age eligibility requirements. If a household had individuals older than 45 and met the residence criterion, one of them was randomly chosen. If the selected individual was 45 or older, he or she become the main respondent, and his or her spouse was interviewed [48]. In its 2011 and 2015 waves, it collected and analyzed venous blood samples [50] by first performing complete blood count (CBC) analyses at local county health centers and then sending the samples to the research headquarters for assay [50]. The CHARLS was conducted according to the guidelines of the Declaration of Helsinki and the Ethical Review Committee at Peking University (IRB 00001052-11014) approved CHARLS for biomarker sample collection. All participants gave their informed consent for the inclusion in the CHARLS study. Survey design and procedures of data collection are discussed on the CHARLS study’s website (http://charls.pku.edu.cn/en, accessed on 15 January 2021) and data, including biomarkers data, are also publicly available at that website.

The sample used for this present study was restricted to adults aged 60+ for whom detailed demographic, socioeconomic, and biomarker information was available in both waves. The final sample size was 8839, with 3732 and 5107 from the CHARLS 2011 and 2015 waves, respectively. To ensure a nationally representative, we adjusted our analytic results by sampling weight.

### 2.2. Healthy Aging: CHAI Score

Given the multidimensional nature of healthy aging, similar to Wu et al. [51], we formulated our corresponding composite measure as a CHAI score based on six physiologic domains, each empirically documented as a key indicator of mortality and a primary measure of a common age-associated chronic disease [52,53]:

- Systolic blood pressure (SBP): the average value of SBP measured three times at 45 s intervals and then grouped into three categories: 0 = ≤120 mmHg, 1 = 120–140 mmHg, and 2 = >140 mmHg. We designated respondents diagnosed with hypertension or taking anti-hypertensive medications as the unhealthiest group (score = 2) [2,51];
- Pulmonary function: the average of expiratory peak flow (L/min) measured three times in a standing position, with gender-specific terciles grouped into three categories (for males: 0 = ≥320 L/min, 1 = 193–320 L/min, and 2 = ≤193 L/min; for females: 0 = ≥225 L/min, 1 = 153–225 L/min, and 2 = ≤153 L/min) [2,51]. We designated respondents diagnosed with pulmonary disease as the unhealthiest group (score = 2);
- Fasting glucose: classified into three categories: 0 = ≤100 mg/dL, 1 = 100–125 mg/dL, and 2 = ≥125 mg/dL [2,51]. We designated respondents diagnosed with diabetes or taking antidiabetic medication as the unhealthiest group (score = 2);
- Cognitive function: as evaluated by the Telephone Interview for Cognitive Status (TICS), whose validity has been confirmed in different populations, including Chinese [54,55]. CHARLS included two cognition measures: episodic memory and mental intactness. The former was based on respondent ability to immediately repeat back in any order 10 Chinese nouns directly read to them (immediate word recall) and then recall the same list 4 min later (delayed recall). By averaging the number of correct answers for both recall types, we generated a 0 to 10 score for aggregate word recall. The second measure, mental intactness, was based on respondent ability to name the date and day of the week, redraw a formerly shown photo, and perform up to five serial 7 subtractions from 100 [55,56]. First, following Lei and Liu [56], we counted each correct answer to generate a 1 to 11 mental intactness score that reflects fluid and crystallized cognition [57]. Then, similar to Luo et al. [55], we summed the mental intactness and episodic memory scores to generate a 0 to 21 total cognition score, which we then classified into the three categories proposed by Wu et al. [51]: for males: 0 = ≥19, 1 = 14–19, and 2 = ≤14; for females: 0 = ≥17, 1 = 10–17, and 2 = ≤10;
- Kidney function: after evaluating individual kidney function based on estimated glomerular filtration rate (eGFR) [58,59], we categorized the eGFR into three groups based on clinically relevant cutoffs taken [58]: 0 = ≥90 mL/min per 1.73 m^2^, 1 = 60–90 mL/min per 1.73 m^2^ and 2 = <60 mL/min per 1.73 m^2^;
- High-sensitivity C-reactive protein (hsCRP): an hsCRP marker of systematic inflammation [60], which we grouped into three categories: for males: 0 = ≤0.81 mg/L, 1 = 0.81–1.98 mg/L, and 2 = ≥1.98 mg/L; for females: 0 = ≤0.77 mg/L, 1 = 0.77–1.86 mg/L, and 2 = ≥1.86 mg/L [2,51].

Using the summed scores for these 6 domains, we derived a CHAI score ranging from 0 (healthiest) to 12 (unhealthiest) for use in our analysis.

### 2.3. Housing Poverty Variables

To evaluate housing poverty, we focused on three dimensions: housing quality poverty, housing quantity poverty, and a combination of the two, each defined as follows: Housing quality poverty was a dummy equal to 1 if the respondent’s house has no potable water, no toilet for sole use, or no kitchen; 0 otherwise. Housing quantity poverty was a dummy equal to 1 if the number of rooms per household member is fewer than 1; 0 otherwise. Housing quality–quantity poverty was a dummy variable equal to 1 if the respondent’s household suffers from both types of poverty; 0 otherwise.

### 2.4. Control Variables

Our models controlled for both individual demographic and socioeconomic characteristics, including age, gender (1 = male, 0 = female), marital status (1 = married, 0 = other), education (measured on a 4-point scale: 1 = illiterate, 2 = primary school, 3 = middle school, and 4 = high school or higher, with illiterate as the reference group) and household expenditure per capita (in CNY). We used household expenditure rather than household income as a proxy of long-term household economic condition [2]. Because both obesity and such risky behaviors as smoking were important predictors of healthy aging [2,61,62,63], we added in an overweight dummy equal to 1 if the body mass index (BMI) is 24 kg/m^2^ or above (0 otherwise); and a smoking dummy equal to 1 if the respondent currently smokes (0 otherwise). Given the empirical evidence of a link between healthy aging and chronic diseases or social activity [2,64,65], we also included a dummy for each. The first was equal to 1 (0 otherwise) if the respondent has been diagnosed with one or more chronic diseases, including hypertension, dyslipidemia, diabetes or high blood sugar, cancer or malignant tumor, chronic lung disease, liver disease; heart disease; stroke; kidney disease; stomach or other digestive disease; emotional, nervous, or psychiatric problems, memory-related disease, arthritis or rheumatism, asthma. The second was equal to 1 (0 otherwise) if the respondent participates in any of the following social activities: interacting with a friend; playing Mahjong, chess, or cards; going to a community club or sporting event; participating in a social group or other club type, belonging to a community-related organization or engaging in voluntary or charity work; and/or attending an educational or training course. Because receiving pension benefits contributes to health both directly (through, for example, better diet, lack of stress, ability to pay for housing) [66] and indirectly through healthcare utilization (e.g., inpatient/outpatient visits, preventive care) [67], we also included a binary variable for whether the respondent is currently receiving any type of nondisability pension (1 = yes, 0 = no). Lastly, given China’s diverse physical geography and its major rural–urban divide in health outcomes [2,61,68], we added in a province dummy to capture possible geographic heterogeneity and a control for current residence location (1 = rural, 0 = urban).

### 2.5. Methods

#### 2.5.1. Psychometric Proprieties Analysis

To assess the psychometric properties of the CHAI score, we focused on five key measures, including acceptability (data completeness and score distribution), internal consistency (the extent to which items in a scale measure the same construct), convergent validity (correlation with other similar measures), discriminative validity (the ability to distinguish between different groups) and precision [69]. Specifically, we assessed acceptability using data quality of fully computable data (generally larger than 95% of fully computable data is acceptable), closeness of mean and median values as well as skewness of the CHAI score distribution (limits: −1 to 1) [70]. Regarding internal consistency, we employed Cronbach’s α coefficient with the minimum accepted value of 0.7 [69,70]. With regards to convergent validity, we followed Daskalopoulou et al. [71] and calculated the Spearman’s rank correlation coefficient (>0.50) between the CHAI score and self-reported health (SRH). Although SRH is subjective, it has been found to be a good predictor of mortality in general [71]. For discriminative validity, we differentiated the CHAI score by different age groups, gender, marital status and education levels. Similar to Rodriguez-Blazquez et al. [69], we then used nonparametric Kruskal–Wallis tests to examine the level of statistical significance of the differences in the CHAI scores across aforementioned subgroups. Finally, we assessed precision of the CHAI score using the standard error of measurement (SEM = standard deviation (SD) X squared root of (1- Cronbach’s α coefficient 0.75)) [69].

#### 2.5.2. Ordinary Least Squares (OLS) Estimation

First, to estimate the association between housing poverty and healthy aging, we applied standard OLS estimation to the following model:

(1) $$HA_{i}=\beta_{0}+\beta_{1}HP_{i}+\beta_{2}X_{i}+\beta_{3}P_{i}+\beta_{4}W_{i}+\varepsilon_{i}$$

where $HA_{i}$ denotes the CHAI score of individual i, and $HP_{i}$ denotes that individual’s housing poverty (represented by a dummy variable for either housing quality poverty, housing quantity poverty, or housing quality–quantity poverty). $X_{i}$ is a vector of individual i’s characteristics, $P_{i}$ is a vector of provincial dummies (with Beijing as the reference), and $\;W_{i}$ is a wave dummy (with 2011 as the reference year). $\beta_{0}\;$ is a constant, while $\beta_{1}$, $\beta_{2}$, $\beta_{3},$ and $\beta_{4}$ are the parameters to be estimated, with $\varepsilon_{i}$ as an error term. Here, captures the effect of housing poverty on an individual’s CHAI score.

#### 2.5.3. UQR

To assess possible heterogeneous effects of housing poverty across the full distribution of CHAI scores, we estimated the UQR model given in Equation (2), which in its simplest form is estimable as an OLS regression on a transformed dependent variable using the recentered influence function (RIF) [72]. Unlike conditional quantile regression, whose identifying covariate impacts the dependent variable’s conditional quantiles, UQR explores the partial effects of its unconditional quantiles [72]:

(2) $$RIF\left(HA_{i};\;Q_{\tau },\;F_{HA}\right)=\delta_{i}HP_{i}+\varphi_{i}X_{i}+\sigma_{i}P_{i}+\theta_{i}W_{i}+\omega_{i\;}$$

where $Q_{\tau }$ denotes the τ*th* quantile of the outcome cumulative distribution $F_{HA}$. $HA_{i}$ and $X_{i}$ follow the same logic as in Equation (1); $\delta_{i}$, $\varphi_{i}$ and $\sigma_{i}$ are the parameters to be estimated; and $\omega_{i}$ is an error term. RIF in Equation (2) is thus

(3) $$RIF\left(HA_{i};\;Q_{\tau },\;F_{HA}\right)=Q_{\tau }+(\tau -I\left[HA_{i}\leq Q_{\tau }\right])/f_{Y}\left(Q_{\tau }\right)$$

where the probability distribution function of variable $HA_{i}$ is $f_{HA}$, and $I\left[HA_{i}\leq Q_{\tau }\right]$ represents the indicator function for whether the CHAI score is small or equal to the *τth* quantile of $F_{HA}$. Similar to Jolliffe [73], we used bootstrapping with 500 replications to obtain unbiased results for the variance-covariance matrix of the parameter estimates.

#### 2.5.4. BO Decomposition

To better understand the contribution of certain socioeconomic, demographic, and health behavioral factors to the healthy aging gap, we used mean-based BO decomposition to account for CHAI score differences between the 2011 and 2015 waves. Hence, we assumed that the nexus between these factors and healthy aging is linear and additive. Two advantages of BO decomposition over regression analysis are its ability to (1) quantify the individual contribution of specific determinants for the CHAI score gap and (2) quantify distributional differences in the factors that explain this gap while also determining the differences in their effects [74]. We decomposed the total difference in mean CHAI score as follows:

(4) $${\bar{Y}}^{2015}-{\bar{Y}}^{2011}=\left({\bar{X}}^{2015}-{\bar{X}}^{2011}\right){\hat{\beta }}^{2015}+{\bar{X}}^{2011}\left({\hat{\beta }}^{2015}-{\hat{\beta }}^{2011}\right)\;$$

where ${\bar{X}}^{i}$ is a vector of the averaged values of the independent variables and ${\hat{\beta }}^{i}$ is a vector of the coefficient estimates for wave i (here, i = 2011, 2015). In Equation (4), the first right-hand term denotes the contribution from distributional differences in the determinant of X, while the second represents that from the differences in every determinant’s effects, thereby identifying all the potential effects of differences in unobserved factors. The contribution of each separate determinant is given by the average change in function when the variable of interest is changed but all others are kept constant.

#### 2.5.5. Recentered Influence Function Regression (RIFR) Decomposition

Because covariate versus coefficient contributions may vary between the median and the tails of the CHAI scores, we adopted a two-step RIFR decomposition to identify the contributions of determinants at different quantiles of the unconditional marginal distribution of the CHAI score distribution. First, after using Equation (3) to derive an RIF at each quantile τ of the CHAI score distribution, we estimated the coefficient on X for each quantile in waves 2011 and 2015 by regressing the RIF on X:

(5) $$Q_{wave,\;\tau }=E_{X}\left[E\left[\hat{RIF}\left(HA;Q_{HA,\;\tau }\right)|X_{wave}\right]\right]=E\left[X_{wave}\right]{\hat{\theta }}_{wave,\tau }$$

where $Q_{wave,\;\tau }$ is the unconditional τth quantile of the CHAI score for waves 2011 and 2015, respectively; and ${\hat{\theta }}_{wave,\tau }$ is the coefficient of the UQR, which captures the marginal effect of a change in the distribution of X on the unconditional quantile of the CHAI score.

Next, we applied BO decomposition to various quantiles (in our case, 25%, 50%, and 75%) calculated by the RIFR:

(6) $${\hat{\Delta }}_{HA}^{\tau }=\left[\hat{RIF}\left(HA_{2015};Q_{2015,\;\tau }\right)\right]-\left[\hat{RIF}\left(HA_{2011};Q_{2011,\;\tau }\right)\right]$$

(7) $${\hat{\Delta }}_{HA}^{\tau }=\left({\bar{X}}_{2015}-{\bar{X}}_{2011}\right){\hat{\theta }}_{2015,\tau }+{\bar{X}}_{2011}\left({\hat{\theta }}_{2015,\tau }-{\hat{\theta }}_{2011,\tau }\right)$$

This procedure decomposes the explained and unexplained parts into the contributions of each covariate at the τth quantile in Equation (7), which, in essence, is processed analogously to the BO decomposition in Equation (4). The decomposition results from both mean-based BO and unconditional quantile-based RIFR should be interpreted as association rather than causality.

## 3. Results

### 3.1. Descriptive Statistics

**Figure 1:**
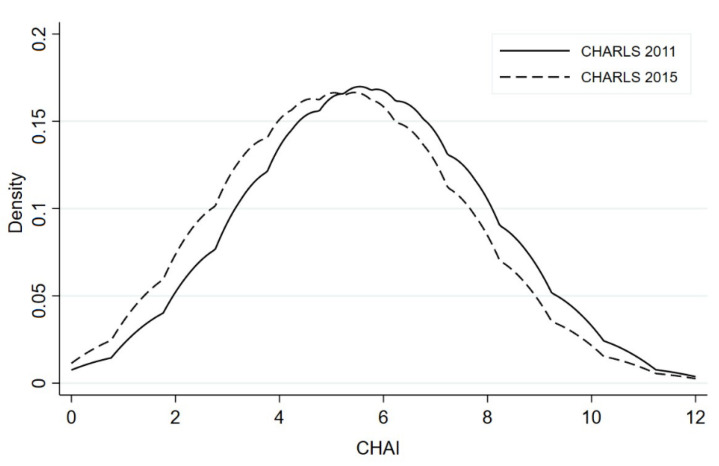
CHAI kernel density by survey year. Notes: Kolmogorov–Smirnov test *p* value: combined K-S = 0.106, *p* value = 0.000.

The mean age of the full sample was approximately 67.7, with around half being males and a majority of respondents married (79.8%) and resident in rural areas (64.8%). The predominant educational levels were illiterate and primary school, at 55.9% and 25.3%, respectively. Across the study period, as Table 1 showed, the average CHAI score for this population decreased from 5.7 in 2011 to 5.2 in 2015, implying a significant improvement in healthy aging, a pattern confirmed in Figure 1 by yearly stratification of the kernel CHAI score densities. At the same time, although the rates of housing quality poverty, quantity poverty, and quality–quantity poverty—at 48.8%, 39.7%, and 21.4%, respectively—suggested a large share of older Chinese experiencing housing poverty, the respective measures represented declines during the period of 11.4, 12.7, and 11.2 pp, implying an improvement in housing poverty over a relatively short amount of time. In terms of health, the rates of chronic diseases and overweight rose over the 2011–2015 period by 8.6 and 6.0 pp, respectively, although physical and social activity also increased by 3 and 2.5 pp and the prevalence of smoking declined by 2.8 pp. Additionally, as in previous studies using the CHARLS data [31,75,76,77], the rate of pension coverage increased sharply from 44% in 2011 to 74% in 2015. The translog household expenditure per capita also significantly rose from 8.6 to 9.1 during the same period.

### 3.2. Analysis of Psychometric Properties

Results of psychometric properties of the CHAI score are shown in Table A1. Regarding acceptability, 100% of the CHAI score were computable. The difference between mean and median of the CHAI score was 0.41, which was quite small. The mean–median discrepancy was much smaller for all six items of the CHAI score. The skewness of the CHAI score was 0.11, which was located within the limit range of −1 to 1. In general, acceptability was satisfactory. For internal consistency, Cronbach’s α coefficient of the CHAI score was 0.71 and item-total corrected correlation ranged from 0.70 to 0.79, which was above the minimum accepted value of 0.7. This means that the internal consistency of the CHAI score was adequate. With regards to convergent validity, the Spearman’s rank correlation coefficient was 0.61 (>0.5). This suggests that the CHAI and SRH had satisfactory convergent validity. In terms of discriminative validity, we found that younger adults, women, the married and higher educated had lower CHAI scores (*p* < 0.0001). For precision of the CHAI score, the SEM was 0.02, showing satisfactory precision.

### 3.3. Impact of Housing Poverty on Healthy Aging

#### 3.3.1. OLS Estimates

According to the OLS results for the housing poverty impact on CHAI, housing quality poverty was significantly and positively associated with a 0.099 increase in the CHAI score (Table 2, column 1), while housing quantity poverty (housing quality–quantity poverty) was positively correlated with a 0.043 (0.096) increase (columns 2 and 3). These findings suggested that, regardless of type, housing poverty is positively associated with a decrease in healthy aging. In addition, we also uniformly found that household expenditure per capita was negatively associated with the CHAI score, with −0.112, −0.115 and −0.112 for housing quality poverty, housing quantity poverty and housing quality–quantity poverty, respectively.

With respect to demographic, socioeconomic, and behavioral characteristics, our findings mirrored those of Nie et al. [2] and Wu et al. [51] for China, and Sanders et al. [65] for the US. Older, male, overweight, rural residents who smoke and have chronic disease were more prone to higher CHAI scores (see Appendix A Table A2), while respondents who were married or cohabiting, better educated, regular participants in social activities, and pension recipients score lower.

#### 3.3.2. RIFR Estimates

Because OLS estimates focus on the effect of explanatory variables at the mean of the CHAI score’s conditional distribution, we used RIFR to estimate the heterogeneous effects of housing poverty on the score’s entire distribution. As Table 3 showed, housing quality poverty was uniformly and positively associated with an increase in the CHAI score at all three selected percentiles. The magnitudes, however, varied, with the largest effect at the upper end of the CHAI distribution (25th: 0.124; 50th: 0.03 and 75th: 0.141; columns 1, 4 and 7). Housing quantity poverty was also positively linked to an increase in CHAI score at all three percentiles but only significantly at lower scores (25th: 0.131, column 2). The combined housing quality–quantity poverty, in contrast, was significantly positively correlated with the CHAI score over its entire distribution, although its magnitude was again largest at the upper end (25th: 0.116; 50th: 0.037 and 75th: 0.138; columns 3, 6 and 9) (for detailed results, see Appendix A Table A2). Taken together, these findings suggested that although housing poverty in all three forms is linked with an overall decline in healthy aging, the unhealthiest older adults may be the most vulnerable, especially in terms of housing quality and quality–quantity poverty. They further implied that heterogeneity in the housing poverty-healthy aging relation exists across the entire distribution, something that mean-based regression such as OLS cannot capture. We also found that household expenditure per capita was significantly and negatively correlated with the CHAI score except for the upper end of the CHAI distribution (though estimated coefficients remain negative).

### 3.4. CHAI Score Differences between the 2011 and 2015 CHARLS Waves

#### 3.4.1. BO Decomposition Estimates

To explain the CHAI score differences between the 2011 and 2015 waves, we reported the conventional BO decomposition results and contributions of the explained (covariates) and unexplained (coefficients) effects as they pertain to the relative contributions of the three housing poverty measures to improving healthy aging over this period (see Table 4). For housing quality, quantity, and quantity–quality poverty, the contributions of the explained part were 31.77%, 31.13%, and 31.77%, respectively (see columns 2, 4 and 6), with housing quality poverty explaining approximately 8% of the improvement in the average CHAI score (column 2). Housing quantity poverty then accounted for around 4% (column 4), while housing quality–quantity poverty explained approximately 7% (column 6). Household expenditure per capita was the most important contributor, accounting for 40.94%, 43.15%, and 40.94% of the explained part in three types of housing poverty.

#### 3.4.2. The RIFR Decomposition Estimates

The results of the RIFR decomposition are presented in Table 5. Several key findings are worth to highlighting: First, the contributions of the explained part to the improvements in the healthy aging ranged from 32.72 to 37.24%. Second, the contribution of housing quality poverty to the explained component increased along the CHAI distribution, from 7.18% at 25th quantile to 14.04% at the median and 23.03% at 75th quantile (see columns 1, 4 and 7). Third, the relative contribution of housing quantity poverty to the explained part ranged from 7.18% at 25th quantile to 8.09% at the median and 9.86% at 75th quantile (see columns 2, 5 and 8). Fourth, the relative contribution of housing quality and quantity poverty to the explained part were more pronounced at the median and the upper distribution of the CHAI score (25th: 7.18%; 50th: 16.11% and 75th: 20%; see columns 3, 6 and 9). Finally, household expenditure per capita made the largest contribution to the 2011–2015 CHAI score difference, ranging from 50.28–50.82%, 60.55–67.05%, and 36.67–44.37% for three types of housing poverty, respectively.

Taken together, the BO and RIFR decomposition results implied that, although household expenditure served as the dominant contributor, housing poverty still played a relatively important role in improving healthy aging in China, especially at the right tail of the CHAI distribution. However, the unexplained portion of the 2011/2015 CHAI score gap remained substantial at 68–69% and 63–67% respectively.

## 4. Discussion

Although a large body of literature examined the housing–health relation, little is known about how housing poverty affects healthy aging in non-Western countries, particularly China. This study thus innovatively combined mean-based BO decomposition with UQR to assess the relative contribution of both qualitative and quantitative housing poverty to 2011–2015 changes in healthy aging outcomes as proxied by a composite CHAI score based on biomarker data (CHARLS 2011 and 2015). This addition of UQR allowed identification of housing poverty’s potential heterogeneous effects along the entire CHAI distribution.

Overall, our analysis of psychometric properties of the CHAI score, including acceptability, internal consistency, convergent validity, discriminative validity and precision, confirmed that the CHAI score displayed good acceptability and reliability as well as adequate validity and precision. Although the 2011–2015 decrease in CHAI score (from 5.69 to 5.20) suggested an improvement in healthy aging, with a substantial simultaneous decline in housing poverty, the continuing prevalence of its qualitative, quantitative, and combined forms (at 48.8%, 39.7%, and 21.4%, respectively) pinpointed it as an ongoing problem for a large share of older Chinese. This prevalence was of particular concern given our finding of an association between all three housing poverty measures and deteriorating health in aging. This latter confirmed a previously documented link between poor housing conditions and a wide range of health issues, including respiratory infections, asthma, lead poisoning, injuries, and mental illness [78,79]. One possible explanation is that housing poverty frequently involves lack of access to clean, safe potable water [80,81,82], whose pollution risk rises with increased contamination by human waste, nutrients, and/or chemicals [80]. At the same time, whereas houses with adequate kitchens and bath facilities can lead to improved diets and personal hygiene, both of which improve health outcomes [83,84], the overcrowding common in housing quantity poverty facilitates transmission of tuberculosis and respiratory infections [79]. In general, therefore, although housing poverty affected healthy aging differently across the CHAI distribution, it particularly affected older adults in poor health.

We also found household expenditure to be significantly associated with healthy aging among older Chinese adults and made the largest contribution to the improvement in healthy aging over time. One possible explanation is that, as stated before, household expenditure serves as the proxy of long-term household economic condition that guarantees the accessibility to a range of health-enhancing behaviors such as good diet, better health knowledge and health services. In addition, respondents who were married or cohabiting, better educated, regular participants in social activities, and pension recipients score lower. These observations supported the literature on obesity, health-related behaviors, and healthy aging or longevity, which showed that overweight, smoking, and chronic disease were detrimental to healthy aging [61,62,63,68]. We also confirmed the important role of higher education as a positive predictor for healthy aging [4], possibly because it leads to better socioeconomic status via more advantageous career paths and higher income levels, which are also linked to healthy aging [85]. Another possible education benefit is general knowledge (particularly, medical knowledge) that facilitates greater health consciousness and preventive action [86,87,88]. Prior studies for China also documented education as an important predictor for improved cognitive ability [4,56].

Admittedly, the unexplained portion of the 2011–2015 CHAI score gap remains substantial (68–69% and 63–67% in the BO and RIFR decompositions, respectively); however, the BO decomposition of the three housing poverty measures clearly explained 4–8% of the 2011–2015 CHAI score difference. It also showed the relative contributions of housing quality and housing quantity poverty to be more pronounced at the median and upper CHAI score, where clinical risks are generally focused.

Our study has several limitations. First, our biomarkers data, especially in the 2011 CHARLS, may suffer from measurement biases. This is attributable to the fact that in the baseline survey, blood collection lagged the survey and the shipping temperature for certain batches of blood was not optimal [50]. However, the quality of biomarkers was improved greatly because the blood samples in the 2015 CHARLS occurred simultaneously with the interviews and the shipping temperature from study sites to the central storage location was strictly controlled and monitored [50]. Second, we assessed how changes of housing poverty affect the CHAI score within a relatively short time period (2011 to 2015). More evidence is needed to explore long-term effects of housing quality and quantity poverty on healthy aging, which will become possible as more waves of biomarker data are available. Finally, although we have attempted to control for confounders of healthy aging when identifying the role of housing poverty in the changes of healthy aging, both decomposition techniques (mean-based BO and unconditional quantile-based) decompose a difference without assessing causality. Therefore, assessing causality in the linkage between housing poverty and healthy aging is one interesting research area that needs further investigation.

## 5. Conclusions

In conclusion, the association between housing poverty and CHAI is independent of household expenditure. Given that household expenditure plays a key role in healthy aging, increasing retirement income may be an effective way to boost healthy aging. In addition, the improvement in healthy aging can be enhanced with better education, in particular, health literacy. China’s “Basic Healthcare and Health Promotion Law” (enacted on 1 June 2020) would integrate health education into the national education system and improve healthy literacy for older adults [2]. Universities for older adults at the community level may play a pivotal role in this aspect. Since housing poverty has more pronounced impacts at the upper CHAI score, where the clinical risk is more prevalent, interventions for health conditions should be targeted at this vulnerable group. Because improved housing conditions boost healthy aging, housing amelioration initiatives may offer an effective solution for augmenting healthy aging in China. Improvement to flushing toilets, access to potable water and the availability of a separate kitchen requires particular attention. Furthermore, since high-density congested housing has a negative impact on healthy aging, more attention can also be paid to improvements in the available space for older people. Especially at an institutional level, the government may extend the housing policy from a homeownership scheme to a housing upgrading scheme by improving housing conditions. This goal can also be greatly advanced by government provision of effective controls and interventions for chronic diseases and overweight; the promotion of healthy lifestyles, especially cessation of smoking; and the facilitation of engagement in both physical and social activities. For example, encouraging health interventions, plans, and actions such as “Healthy China Action (2019–2030)” would pay special attention to older adults and promote their healthy eating habits and mitigate disease risk. In addition, regarding the local government, providing access to key community services such as green spaces, parks and public recreation places with sports facilities would provide a more livable and inclusive environment, thereby enhancing the participation in both physical and social activities of older people. As China will continue to rapidly urbanize and age, planning sustainable housing provisions with better housing conditions will improve healthy aging and thus give rise to significant economic benefits in the long run.

## Figures and Tables

**Table 1:** Descriptive statistics for Chinese adults aged 60+: CHARLS 2011 and 2015.

Variable	Total		2011	2015	Mean Difference
	Mean	SD	Mean	Mean	
Dependent variable					
CHAI (0–12)	5.408	2.057	5.694	5.200	−0.494 ***
Independent variable					
Housing poverty					
Housing quality poverty (1 = yes, 0 = no)	0.488	0.500	0.554	0.440	−0.114 ***
No potable water	0.340	0.474	0.427	0.277	−0.150 ***
No toilet for sole use	0.247	0.431	0.281	0.222	−0.059 ***
No kitchen	0.086	0.280	0.090	0.083	−0.007 **
Housing quantity poverty (1 = yes, 0 = no)					
Rooms per household member <1	0.397	0.489	0.471	0.344	−0.127 ***
Housing quality–quantity poverty (1 = yes, 0 = no)	0.214	0.410	0.279	0.167	−0.112 ***
Control variable					
Age group	67.696	6.227	67.714	67.682	−0.032
60–64	0.384	0.486	0.398	0.374	−0.024 **
65–69	0.280	0.449	0.262	0.293	0.031 ***
70–74	0.180	0.384	0.175	0.183	0.008
75–79	0.105	0.307	0.112	0.100	−0.012 *
≥80	0.051	0.221	0.053	0.050	−0.003
Gender (1 = male, 0 = female)	0.498	0.500	0.501	0.497	−0.004
Marital status (1 = married, 0 = other)	0.798	0.401	0.798	0.798	0.000
Current residence (1 = rural, 0 = urban)	0.648	0.478	0.656	0.642	−0.014
Educational level	1.686	0.906	1.633	1.724	0.091 ***
Illiterate	0.559	0.497	0.579	0.545	−0.034 ***
Primary school	0.253	0.435	0.258	0.250	−0.008
Middle school	0.130	0.336	0.113	0.141	0.028 ***
High school or higher	0.058	0.233	0.049	0.064	0.015 ***
Chronic disease (1 = yes, 0 = no)	0.838	0.369	0.788	0.874	0.086 ***
Overweight (BMI ≥ 24 kg/m^2^)	0.404	0.491	0.369	0.429	0.060 ***
Smoking (1 = yes, 0 = no)	0.290	0.454	0.306	0.278	−0.028 ***
Physical activity (1 = yes, 0 = no)	0.140	0.347	0.122	0.152	0.030 ***
Social activity (1 = yes, 0 = no)	0.458	0.498	0.443	0.468	0.025 **
Pension (1 = yes, 0 = no)	0.612	0.487	0.435	0.740	0.305 ***
Log (HH expenditure per capita)	8.899	0.039	8.590	9.137	0.547 ***
Obs.	8839		3732	5107	

Notes: CHAI, the Chinese Healthy Aging Index, on which higher values denote worse healthy aging. The significance of the mean difference is based on independent *t* tests. * *p* ≤ 0.1, ** *p* ≤ 0.05, *** *p* ≤ 0.01.

**Table 2:** OLS estimates of housing poverty’s impact on CHAI among Chinese adults aged 60+: CHARLS 2011 and 2015.

Variable	OLS		
Housing Poverty	(1)	(2)	(3)
Housing quality poverty	0.099 **		
	(0.044)		
Housing quantity poverty		0.043	
		(0.041)	
Housing quality–quantity poverty			0.096 *
			(0.053)
HH expenditure per capita	−0.112 ***	−0.115 ***	−0.112 ***
	(0.025)	(0.025)	(0.025)
Year dummy	YES	YES	YES
Province dummy	YES	YES	YES
Observations	8839	8839	8839
*R* ^2^	0.253	0.253	0.253

Notes: The dependent variable is the CHAI score; controls in the explained part include age group, gender, education, marital status, current residence (rural vs. urban), smoking, chronic disease, overweight, participation in social and physical activity, pension benefits, household expenditure per capita and year and province dummies. Standard errors are reported in parentheses. * *p* ≤ 0.1, ** *p* ≤ 0.05, *** *p* ≤ 0.01.

**Table 3:** Unconditional quantile regression of housing poverty’s impact on CHAI among Chinese adults aged 60+: CHARLS 2011 and 2015 (based on Appendix A Table A3).

Variables	25th			50th			75th		
Housing Poverty	(1)	(2)	(3)	(4)	(5)	(6)	(7)	(8)	(9)
Housing quality poverty	0.124 **			0.033 **			0.141 **		
	(0.052)			(0.048)			(0.060)		
Housing quantity poverty		0.131 ***			0.005			0.060	
		(0.049)			(0.046)			(0.057)	
Housing quality–quantity poverty			0.116 *			0.037 *			0.138 *
			(0.063)			(0.059)			(0.073)
HH expenditure per capita	−0.127 ***	−0.124 ***	−0.128 ***	−0.130 ***	−0.133 ***	−0.130 ***	−0.048	−0.053	−0.049
	(0.030)	(0.030)	(0.030)	(0.028)	(0.028)	(0.028)	(0.035)	(0.035)	(0.035)
Year dummy	YES	YES	YES	YES	YES	YES	YES	YES	YES
Province dummy	YES	YES	YES	YES	YES	YES	YES	YES	YES
Observations	8839	8839	8839	8839	8839	8839	8839	8839	8839
*R* ^2^	0.116	0.116	0.116	0.158	0.158	0.158	0.151	0.150	0.151

Notes: The dependent variable is the CHAI score; controls in the explained part include age groups, gender, education, marital status, current residence (rural vs. urban), smoking, chronic disease, overweight, participation in social and physical activity, pension benefits, household expenditure per capita and year and province dummies. Standard errors are reported in parentheses. * *p* ≤ 0.1, ** *p* ≤ 0.05, *** *p* ≤ 0.01.

**Table 4:** Blinder–Oaxaca decomposition of determinants for the changes in CHAI among Chinese adults aged 60+: CHARLS 2011 and 2015 (contributions based on Appendix A Table A4, %).

		Contribution (%)		Contribution (%)		Contribution (%)
	(1)	(2)	(3)	(4)	(5)	(6)
CHAI: CHARLS 2011	5.685 ***		5.685 ***		5.685 ***	
CHAI: CHARLS 2015	5.216 ***		5.216 ***		5.216 ***	
Total difference	−0.469 ***		−0.469 ***		−0.469 ***	
Explained part	−0.149 ***	31.77	−0.146 ***	31.13	−0.149 ***	31.77
Unexplained part	−0.321 ***	68.44	−0.324 ***	69.08	−0.321 ***	68.44
Explained part						
Housing quality poverty	−0.012 ***	8.05				
	(0.006)					
Housing quantity poverty			−0.006	4.11		
			(0.007)			
Housing quality–quantity poverty					−0.011 **	7.38
					(0.007)	
HH expenditure per capita	−0.061 **	40.94	−0.063 **	43.15	−0.061 **	40.94
	(0.020)		(0.020)		(0.020)	

Notes: The dependent variable is the CHAI score; controls in the explained part include age group, gender, education, marital status, current residence (rural vs. urban), smoking, chronic disease, overweight, participation in social and physical activity, pension benefits, household expenditure per capita and year and province dummies. Standard errors are reported in parentheses. ** *p* ≤ 0.05, *** *p* ≤ 0.01.

**Table 5:** The RIF decomposition of determinants for the changes of CHAI among the elderly aged 60+, CHARLS 2011 and 2015 (contributions based on Appendix A Table A5, %).

Quantiles	25th			50th			75th		
	(1)	(2)	(3)	(4)	(5)	(6)	(7)	(8)	(9)
CHAI: CHARLS 2011		4.662			6.145			7.580	
CHAI: CHARLS 2015		4.176			5.628			7.147	
Total difference		−0.486 ***			−0.517 ***			−0.434 ***	
Explained part	37.24	37.24	37.24	34.43	33.46	34.82	35.02	32.72	34.56
Unexplained part	62.76	62.76	62.76	65.57	66.54	65.18	64.75	67.05	65.44
Explained part									
Housing quality poverty	7.18			14.04			23.03		
Housing quantity poverty		7.18			8.09			9.86	
Housing quality and quantity poverty			7.18			16.11			20.00
HH expenditure per capita	50.83	50.28	50.28	62.91	67.05	60.56	36.84	44.37	36.67

Notes: The dependent variable is the CHAI score. Controls in the explained part also include age groups, gender, education, marital status, current residence (rural vs. urban), smoking, having chronic disease, overweight, participation in social activity and physical activity, pension benefits, household expenditure per capita, year and provincial dummies. *** *p* ≤ 0.01.

## Data Availability

The datasets used and analyzed during the current study are available in http://forum.charls.pku.edu.cn/, accessed on 15 January 2021.

## Appendix A

**Table A1:** The analysis of psychometric properties of the CHAI score.

	Acceptability and Internal Consistency						
	Fully Computable (%)	Mean	Median	SD	Skewness	Observed Min.–Max.	ITCC (Cronbach’s α)
CHAI score	100.0	5.41	5.00	2.06	0.11	0–12	0.71
1. Systolic blood pressure	100.0	1.09	1.00	0.80	−0.16	0–2	0.78
2. Pulmonary function	100.0	0.85	1.00	0.80	0.28	0–2	0.78
3. Fasting glucose	100.0	0.90	1.00	0.79	0.18	0–2	0.79
4. Cognitive function	100.0	0.58	0.00	0.69	0.78	0–2	0.71
5. Kidney function	100.0	0.93	1.00	0.45	−0.30	0–2	0.71
6. High-sensitivity C-reactive protein	100.0	1.07	1.00	0.81	−0.12	0–2	0.70
	Convergent validity						
Spearman’s rank correlation coefficient							
CHAI score and Self-reported health (SRH)	0.61 ***						
	Discriminative validity						
			Mean	SD	*p*-value		
Age					0.0001		
60–64 years			4.71	1.93			
65–69 years			5.24	1.85			
70–74 years			5.96	2.00			
75–79 years			6.55	1.90			
80 years and over			7.34	1.83			
Gender					0.0001		
Women			5.32	2.14			
Men			5.52	2.01			
Marital status					0.0001		
Others			6.16	2.11			
Married			5.21	2.02			
Education					0.0001		
No education			5.83	2.06			
Primary school			5.37	2.02			
Middle school			4.52	1.93			
High school or higher			4.73	1.95			
	Precision						
Standard error of measurement (SEM)	0.02						

Notes: For discriminative validity, *p* values are based on Kruskal–Wallis tests. ITCC, item total corrected correlation. *** *p* ≤ 0.01.

**Table A2:** OLS estimates of housing poverty’s impact on CHAI scores among Chinese adults aged 60+: CHARLS 2011 and 2015.

Variable	OLS		
Housing Poverty	(1)	(2)	(3)
Housing quality poverty	0.099 **		
	(0.044)		
Housing quantity poverty		0.043	
		(0.041)	
Housing quality–quantity poverty			0.096 *
			(0.053)
Age group			
65–69	0.487 ***	0.492 ***	0.490 ***
	(0.049)	(0.049)	(0.049)
70–74	1.113 ***	1.117 ***	1.115 ***
	(0.056)	(0.056)	(0.056)
75–79	1.806 ***	1.808 ***	1.806 ***
	(0.066)	(0.066)	(0.066)
≥80	2.412 ***	2.412 ***	2.412 ***
	(0.087)	(0.087)	(0.087)
Male	0.491 ***	0.491 ***	0.491 ***
	(0.047)	(0.047)	(0.047)
Married	−0.394 ***	−0.401 ***	−0.404 ***
	(0.050)	(0.051)	(0.051)
Rural resident	0.032	0.006	0.015
	(0.047)	(0.046)	(0.046)
Education: primary school	−0.388 ***	−0.392 ***	−0.389 ***
	(0.050)	(0.050)	(0.050)
Education: middle school	−0.917 ***	−0.926 ***	−0.921 ***
	(0.063)	(0.063)	(0.063)
Education: high school or higher	−0.876 ***	−0.888 ***	−0.882 ***
	(0.081)	(0.081)	(0.081)
Chronic disease	0.362 ***	0.364 ***	0.363 ***
	(0.054)	(0.054)	(0.054)
Overweight (BMI ≥ 24 kg/m^2^)	0.691 ***	0.687 ***	0.690 ***
	(0.042)	(0.041)	(0.042)
Smoking	0.148 ***	0.153 ***	0.151 ***
	(0.050)	(0.050)	(0.050)
Social activity	−0.225 ***	−0.227 ***	−0.226 ***
	(0.040)	(0.040)	(0.040)
Pension	−0.195 ***	−0.195 ***	−0.194 ***
	(0.043)	(0.043)	(0.043)
Log (HH expenditure per capita)	−0.112 ***	−0.115 ***	−0.112 ***
	(0.025)	(0.025)	(0.025)
Year dummy	YES	YES	YES
Province dummy	YES	YES	YES
Observations	8839	8839	8839
Adj. *R*^2^	0.253	0.253	0.253

Notes: 95% confidence internals are reported in parentheses. * *p* ≤ 0.1, ** *p* ≤ 0.05, *** *p* ≤ 0.01.

**Table A3:** Unconditional quantile regression of housing poverty’s impact on CHAI scores among Chinese adults aged 60+: CHARLS 2011 and 2015.

Variable	25th			50th			75th		
Housing Poverty	(1)	(2)	(3)	(4)	(5)	(6)	(7)	(8)	(9)
Housing quality poverty	0.124 **			0.033 **			0.141 **		
	(0.052)			(0.048)			(0.060)		
Housing quantity poverty		0.131 ***			0.005			0.060	
		(0.049)			(0.046)			(0.057)	
Housing quality–quantity poverty			0.116 *			0.037 *			0.138 *
			(0.063)			(0.059)			(0.073)
Age group									
65–69	0.467 ***	0.476 ***	0.470 ***	0.457 ***	0.457 ***	0.457 ***	0.390 ***	0.396 ***	0.393 ***
	(0.060)	(0.060)	(0.060)	(0.056)	(0.056)	(0.056)	(0.070)	(0.070)	(0.070)
70–74	0.907 ***	0.920 ***	0.910 ***	1.002 ***	1.002 ***	1.003 ***	1.077 ***	1.083 ***	1.080 ***
	(0.067)	(0.068)	(0.067)	(0.063)	(0.063)	(0.063)	(0.078)	(0.079)	(0.078)
75–79	1.166 ***	1.172 ***	1.165 ***	1.397 ***	1.396 ***	1.397 ***	2.071 ***	2.072 ***	2.071 ***
	(0.080)	(0.080)	(0.080)	(0.074)	(0.074)	(0.074)	(0.093)	(0.093)	(0.093)
≥80	1.353 ***	1.356 ***	1.353 ***	1.718 ***	1.717 ***	1.718 ***	2.689 ***	2.687 ***	2.688 ***
	(0.105)	(0.106)	(0.106)	(0.098)	(0.098)	(0.098)	(0.122)	(0.123)	(0.123)
Male	0.543 ***	0.540 ***	0.542 ***	0.494 ***	0.494 ***	0.494 ***	0.396 ***	0.395 ***	0.396 ***
	(0.057)	(0.057)	(0.057)	(0.053)	(0.053)	(0.053)	(0.066)	(0.066)	(0.066)
Married	−0.293 ***	−0.310 ***	−0.305 ***	−0.372 ***	−0.372 ***	−0.376 ***	−0.403 ***	−0.413 ***	−0.418 ***
	(0.061)	(0.061)	(0.061)	(0.056)	(0.057)	(0.057)	(0.071)	(0.071)	(0.071)
Rural resident	0.024	0.010	0.003	0.087 *	0.080	0.082 *	0.018	0.053	0.042
	(0.054)	(0.052)	(0.052)	(0.050)	(0.049)	(0.049)	(0.062)	(0.061)	(0.061)
Education: primary school	−0.374 ***	−0.378 ***	−0.376 ***	−0.344 ***	−0.346 ***	−0.345 ***	−0.452 ***	−0.459 ***	−0.454 ***
	(0.060)	(0.060)	(0.060)	(0.056)	(0.056)	(0.056)	(0.070)	(0.070)	(0.070)
Education: middle school	−0.984 ***	−0.992 ***	−0.989 ***	−1.066 ***	−1.069 ***	−1.067 ***	−0.788 ***	−0.799 ***	−0.794 ***
	(0.074)	(0.074)	(0.074)	(0.069)	(0.069)	(0.069)	(0.087)	(0.086)	(0.086)
Education: high school or higher	−0.943 ***	−0.952 ***	−0.951 ***	−0.881 ***	−0.885 ***	−0.882 ***	−0.917 ***	−0.932 ***	−0.925 ***
	(0.097)	(0.097)	(0.097)	(0.090)	(0.090)	(0.090)	(0.113)	(0.112)	(0.113)
Chronic disease	0.330 ***	0.333 ***	0.331 ***	0.411 ***	0.411 ***	0.411 ***	0.372 ***	0.376 ***	0.374 ***
	(0.066)	(0.066)	(0.066)	(0.061)	(0.061)	(0.061)	(0.076)	(0.076)	(0.076)
Overweight (BMI ≥ 24 kg/m^2^)	0.598 ***	0.593 ***	0.597 ***	0.581 ***	0.580 ***	0.581 ***	0.581 ***	0.576 ***	0.580 ***
	(0.050)	(0.050)	(0.050)	(0.046)	(0.046)	(0.046)	(0.058)	(0.058)	(0.058)
Smoking	0.092	0.099 *	0.097	0.114 **	0.116 **	0.115 **	0.175 **	0.184 ***	0.180 ***
	(0.060)	(0.060)	(0.060)	(0.056)	(0.056)	(0.056)	(0.070)	(0.070)	(0.070)
Social activity	−0.211 ***	−0.213 ***	−0.213 ***	−0.258 ***	−0.259 ***	−0.259 ***	−0.264 ***	−0.267 ***	−0.266 ***
	(0.048)	(0.048)	(0.048)	(0.045)	(0.045)	(0.045)	(0.056)	(0.056)	(0.056)
Pension	−0.155 ***	−0.156 ***	−0.155 ***	−0.081 *	−0.081 *	−0.081 *	−0.101 *	−0.102 *	−0.100 *
	(0.051)	(0.051)	(0.051)	(0.047)	(0.047)	(0.047)	(0.059)	(0.059)	(0.059)
Household expenditure per capita	−0.127 ***	−0.124 ***	−0.128 ***	−0.130 ***	−0.133 ***	−0.130 ***	−0.048	−0.053	−0.049
	(0.030)	(0.030)	(0.030)	(0.028)	(0.028)	(0.028)	(0.035)	(0.035)	(0.035)
Year dummy	YES	YES	YES	YES	YES	YES	YES	YES	YES
Province dummy	YES	YES	YES	YES	YES	YES	YES	YES	YES
Observations	8839	8839	8839	8839	8839	8839	8839	8839	8839
Adj. *R*^2^	0.116	0.116	0.116	0.158	0.158	0.158	0.151	0.150	0.151

Notes: 95% confidence internals are reported in parentheses. * *p* ≤ 0.1, ** *p* ≤ 0.05, *** *p* ≤ 0.01.

**Table A4:** Blinder–Oaxaca decomposition of the determinants of CHAI score changes among Chinese adults aged 60+: CHARLS 2011 and 2015.

		Contribution (%)		Contribution (%)		Contribution (%)
	(1)	(2)	(3)	(4)	(5)	(6)
CHAI: CHARLS 2011	5.685 ***		5.685 ***		5.685 ***	
CHAI: CHARLS 2015	5.216 ***		5.216 ***		5.216 ***	
Total difference	−0.469 ***		−0.469 ***		−0.469 ***	
Explained part	−0.149 **	31.77	−0.146 **	31.13	−0.149 **	31.77
Unexplained part	−0.321 ***	68.44	−0.324 ***	69.08	−0.321 ***	68.44
Explained part						
Housing quality poverty	−0.012 ***	8.05				
	(0.006)					
Housing quantity poverty			−0.006	4.11		
			(0.007)			
Housing quality–quantity poverty					−0.011 **	7.38
					(0.007)	
Age	−0.048 *	32.21	−0.048 *	32.88	−0.048 *	32.21
	(0.026)		(0.026)		(0.026)	
Gender	0.011	−7.38	0.011	−7.53	0.011	−7.38
	(0.009)		(0.009)		(0.009)	
Married	−0.009	6.04	−0.009	6.16	−0.010	6.71
	(0.006)		(0.006)		(0.006)	
Rural resident	0.001	−0.67	0.000	−0.01	0.000	−0.01
	(0.002)		(0.002)		(0.002)	
Education	−0.042 **	28.19	−0.043 **	29.45	−0.042 **	28.19
	(0.015)		(0.015)		(0.015)	
Chronic disease	0.031 ***	−20.81	0.031 ***	−21.23	0.031 ***	−20.81
	(0.007)		(0.007)		(0.007)	
Overweight (BMI ≥ 24 kg/m^2^)	0.037 **	−24.83	0.037 **	−25.34	0.037 **	−24.83
	(0.013)		(0.013)		(0.013)	
Smoking	0.002	−1.34	0.002	−1.37	0.002	−1.34
	(0.002)		(0.002)		(0.002)	
Social activity	−0.007	4.70	−0.007	4.79	−0.007	4.70
	(0.004)		(0.004)		(0.004)	
Pension	−0.047 ***	31.54	−0.047 ***	32.19	−0.047 ***	31.54
	(0.014)		(0.014)		(0.014)	
Household expenditure per capita	−0.061 **	40.94	−0.063 **	43.15	−0.061 **	40.94
	(0.020)		(0.020)		(0.020)	
Province	−0.004	2.68	−0.004	2.74	−0.004	2.68
	(0.010)		(0.010)		(0.010)	

Notes: 95% confidence internals are reported in parentheses. * *p* ≤ 0.1, ** *p* ≤ 0.05, *** *p* ≤ 0.01.

**Table A5:** RIF decomposition of the determinants of CHAI score changes among Chinese adults aged 60+: CHARLS 2011 and 2015.

		25th			50th			75th	
	(1)	(2)	(3)	(4)	(5)	(6)	(7)	(8)	(9)
CHAI: CHARLS 2011		4.662 ***			6.144 ***			7.580 ***	
		(0.077)			(0.065)			(0.070)	
CHAI: CHARLS 2015		4.176 ***			5.627 ***			7.146 ***	
		(0.055)			(0.051)			(0.062)	
Total difference		−0.486 ***			−0.517 ***			−0.434 ***	
		(0.095)			(0.082)			(0.093)	
Explained part	−0.181 *	−0.181 *	−0.181 *	−0.178 **	−0.173 **	−0.180 **	−0.152 *	−0.142 *	−0.150 *
	(0.072)	(0.074)	(0.072)	(0.065)	(0.065)	(0.064)	(0.066)	(0.067)	(0.066)
Unexplained part	−0.305 *	−0.305 *	−0.305 *	−0.339 ***	−0.344 ***	−0.337 ***	−0.281 **	−0.291 **	−0.284 **
	(0.124)	(0.126)	(0.124)	(0.096)	(0.096)	(0.095)	(0.104)	(0.104)	(0.104)
Explained part									
Housing quality poverty	−0.013 **			−0.025 **			−0.035 **		
	(0.011)			(0.012)			(0.015)		
Housing quantity poverty		−0.013			−0.014			−0.014	
		(0.013)			(0.013)			(0.015)	
Housing quality–quantity poverty			−0.013 **			−0.029 **			−0.030 **
			(0.011)			(0.012)			(0.014)
Age	−0.032 **	−0.032 **	−0.032 **	−0.045 **	−0.044 **	−0.045 **	−0.057 **	−0.057 **	−0.057 **
	(0.018)	(0.018)	(0.018)	(0.024)	(0.024)	(0.024)	(0.030)	(0.030)	(0.030)
Gender	0.011	0.011	0.011	0.002	0.003	0.002	0.008	0.008	0.008
	(0.009)	(0.010)	(0.009)	(0.003)	(0.004)	(0.004)	(0.007)	(0.007)	(0.007)
Married	−0.011	−0.011	−0.011	−0.009	−0.009	−0.010	−0.008	−0.008	−0.008
	(0.007)	(0.007)	(0.007)	(0.006)	(0.006)	(0.006)	(0.006)	(0.006)	(0.006)
Rural resident	0.008	0.007	0.008	0.007	0.005	0.006	0.006	0.004	0.004
	(0.006)	(0.006)	(0.006)	(0.006)	(0.005)	(0.005)	(0.006)	(0.005)	(0.005)
Education	−0.038	−0.039 *	−0.039	−0.037 *	−0.039 *	−0.037 *	−0.036 *	−0.038 *	−0.037 *
	(0.020)	(0.020)	(0.020)	(0.018)	(0.018)	(0.018)	(0.017)	(0.017)	(0.017)
Chronic disease	0.037 **	0.037 **	0.037 **	0.038 ***	0.038 ***	0.038 ***	0.025 *	0.025 *	0.025 *
	(0.012)	(0.012)	(0.012)	(0.010)	(0.011)	(0.010)	(0.011)	(0.011)	(0.011)
Overweight (BMI ≥ 24 kg/m^2^)	0.026 *	0.026 *	0.026 *	0.029 *	0.029 *	0.029 *	0.035 *	0.035 *	0.035 *
	(0.011)	(0.011)	(0.011)	(0.012)	(0.012)	(0.012)	(0.014)	(0.014)	(0.014)
Smoking	0.002	0.002	0.002	0.005	0.005	0.005	0.003	0.003	0.003
	(0.003)	(0.003)	(0.003)	(0.006)	(0.006)	(0.006)	(0.005)	(0.005)	(0.005)
Social activity	−0.009	−0.009	−0.009	−0.009	−0.009	−0.009	−0.009	−0.009	−0.009
	(0.006)	(0.007)	(0.007)	(0.006)	(0.006)	(0.006)	(0.007)	(0.007)	(0.007)
Pension	−0.061 *	−0.060	−0.061 *	−0.018	−0.017	−0.018	−0.025	−0.025	−0.026
	(0.031)	(0.031)	(0.031)	(0.030)	(0.030)	(0.030)	(0.033)	(0.033)	(0.033)
Household expenditure per capita	−0.092 *	−0.092 *	−0.091 *	−0.112 **	−0.116 ***	−0.109 **	−0.056	−0.063	−0.055
	(0.038)	(0.036)	(0.038)	(0.035)	(0.035)	(0.035)	(0.039)	(0.038)	(0.039)
Province	−0.010	−0.010	−0.010	−0.004	−0.005	−0.004	−0.003	−0.004	−0.003
	(0.026)	(0.026)	(0.026)	(0.022)	(0.023)	(0.023)	(0.019)	(0.019)	(0.019)

Notes: 95% confidence internals are reported in parentheses. * *p* ≤ 0.1, ** *p* ≤ 0.05, *** *p* ≤ 0.01.
